# Spontaneous disappearance of common bile duct stones following the insertion of a new dumbbell‐shaped, covered self‐expandable metallic stent in a patient with benign biliary stricture

**DOI:** 10.1002/ccr3.4093

**Published:** 2021-05-04

**Authors:** Yuto Ishizaki, Mitsuru Sugimoto, Tadayuki Takagi, Rei Suzuki, Naoki Konno, Hiroyuki Asama, Yuki Sato, Hiroki Irie, Yoshinori Okubo, Jun Nakamura, Mika Takasumi, Minami Hashimoto, Tsunetaka Kato, Ryoichiro Kobashi, Takuto Hikichi, Hiromasa Ohira

**Affiliations:** ^1^ Department of Gastroenterology, School of Medicine Fukushima Medical University Fukushima Japan; ^2^ Department of Endoscopy Fukushima Medical University Hospital Fukushima Japan

**Keywords:** benign biliary stricture, chronic pancreatitis, common bile duct stones

## Abstract

The new dumbbell‐shaped, covered self‐expanding metallic stent can efficiently remove the choledocholiths in cases with common bile duct (CBD) strictures; moreover, it may potentially prevent a positional displacement and contribute to the better improvement of the CBD stricture.

## Graphical abstract

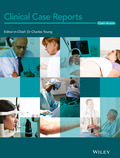

## 1 INTRODUCTION

Endoscopic removal of bile stones is challenging in cases with a distal stricture of the common bile duct. A new dumbbell‐shaped covered self‐expanding metallic stent can efficiently remove the common bile duct stones in cases with stricture of the common bile duct caused by chronic pancreatitis.

Common bile duct (CBD) stones are usually a risk factor for cholangitis; hence, their removal is recommended. Surgical or endoscopic lithotomy is the preferred treatment modality to remove the CBD stones. Endoscopic lithotomy has become the first choice of treatment as it is a minimally invasive procedure.

Removal of CBD stones by endoscopic lithotomy may be challenging in patients with a very large stone size, multiple stones packed closely,^1^, ^2^, ^3^, ^4^, ^5^ a history of digestive tract reconstruction,^6^, ^7^ or a combination of these factors. In addition to these factors, the presence of a CBD stricture also increases the difficulty of endoscopic lithotomy.^8^, ^9^, ^10^ If a distal CBD stricture is narrower than the diameter of the CBD stones, removing the stones becomes further complicated.

We report a case in which the removal of CBD stones was difficult due to a CBD stricture caused by chronic pancreatitis (CP); hence, endoscopic lithotripsy was also challenging. However, the stones were spontaneously removed following the insertion of a covered self‐expanding metallic stent (CSEMS).

## 2 CASE PRESENTATION

**FIGURE 1:**
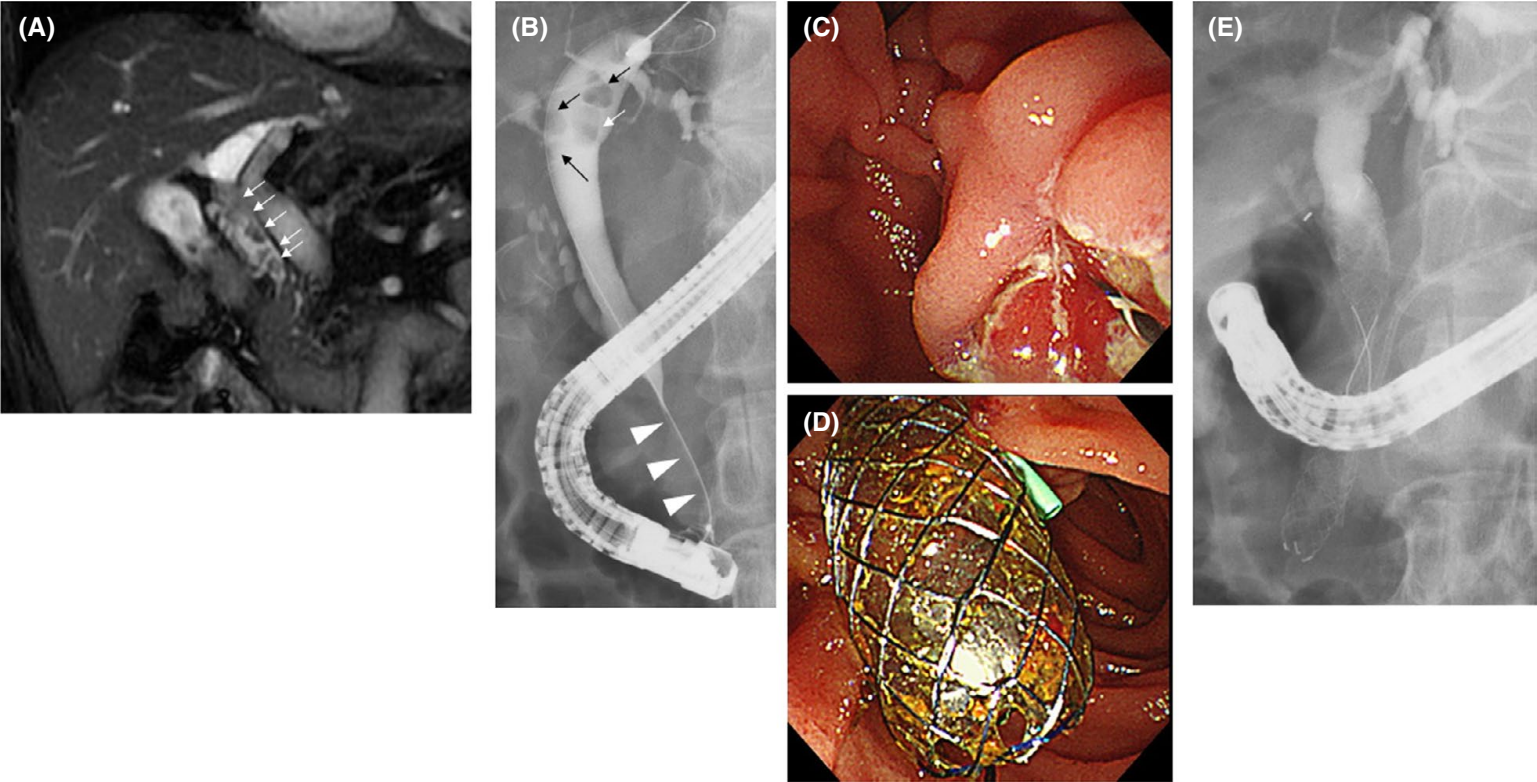
The clinical course until the first endoscopic retrograde cholangiopancreatography (ERCP). A, Common bile duct (CBD) stones (arrow) were detected on magnetic resonance cholangiopancreatography. B, In addition to the CBD stones (arrow), distal CBD stricture (arrowhead) was observed on endoscopic cholangiography. C, After cholangiography, endoscopic sphincterotomy was performed. D, E, A covered self‐expandable metallic stent was placed to dilate the distal CBD stricture after the pancreatic stent was inserted to prevent post‐ERCP pancreatitis

A 65‐year‐old man was admitted to our hospital for a periodic medical examination for CP. He also underwent periodic imaging examinations during follow‐up, for the last 10 years, for a benign biliary stricture caused by CP. Magnetic resonance cholangiopancreatography (MRCP) conducted as a part of these examinations revealed multiple CBD stones (Figure 1A). Contrast‐enhanced computed tomography performed 6 months before the MRCP had not revealed stones in the CBD; however, gallbladder stones were seen. It was presumed that the gallbladder stones had now progressively moved to the CBD. Therefore, we performed an endoscopic therapy for the CBD stones.

**FIGURE 2:**
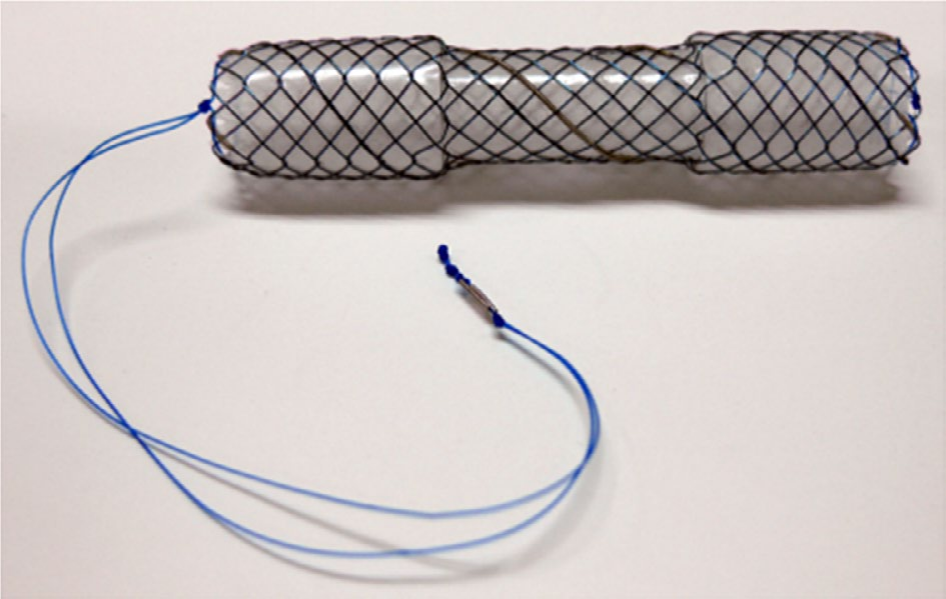
The covered self‐expandable metallic stent (CSEMS) used in this case was the BONASTENT M‐intraductal. The shape of the stent resembles a dumbbell. By pulling a string, the BONASTENT M‐intraductal becomes narrower. Therefore, the CSEMS is easy to remove

An endoscopic cholangiography revealed a stricture of the distal CBD and four CBD stones. The shortest diameter of each of the four stones (6, 5, 4, and 3 mm) was larger than the diameter of the distal CBD lumen (Figure 1B); therefore, endoscopic extraction of the stones was challenging. After endoscopic sphincterotomy (Figure 1C), we attempted to remove the CBD stones using a basket catheter, which is often used for the destruction of stones. However, the movement of the basket catheter was limited by the biliary stricture. Therefore, the basket catheter could only hold a small stone but could not extract them. The distal biliary duct was extremely narrow. Therefore, there was a possibility that the placement of multiple biliary plastic stents would be difficult, or they would not dilate the distal biliary stricture adequately to remove the CBD stones. Additionally, no pancreatic atrophy was observed during the imaging examinations. Due to the risk of injury to the pancreatic parenchyma, we did not perform dilatation with a balloon catheter. Considering all these factors, a CSEMS was placed to dilate the distal CBD stricture (Figure 1D–E). The CSEMS used in this case was BONASTENT M‐intraductal 8 mm 7 cm (Standard Sci Tech). The stent has a dumbbell shape, with an 8 mm diameter at both ends and a 6 mm diameter at the center (Figure 2). The distal tip of the stent was pushed out from the duodenum to prevent proximal migration of the stent. Due to the flared tips of the BONASTENT M‐intraductal, the risk of proximal migration of the stent is reduced. The diameter of the CSEMS (8 mm) was the same as the diameter of the CBD. The length of the distal biliary stricture was 3 cm. A 7 or 6 cm‐long stent had a midportion length of 2 cm, which is the longest BONASTENT M‐intraductal available commercially. In this case, the superior portion of the distal biliary stricture was slightly narrow. Therefore, we selected a longer stent of 7 cm. Before the CSEMS insertion, a 5‐Fr 12 cm straight pancreatic stent was placed in the main pancreatic duct to prevent postendoscopic retrograde cholangiopancreatography pancreatitis.^11^, ^12^, ^13^, ^14^, ^15^, ^16^, ^17^, ^18^, ^19^

**FIGURE 3:**
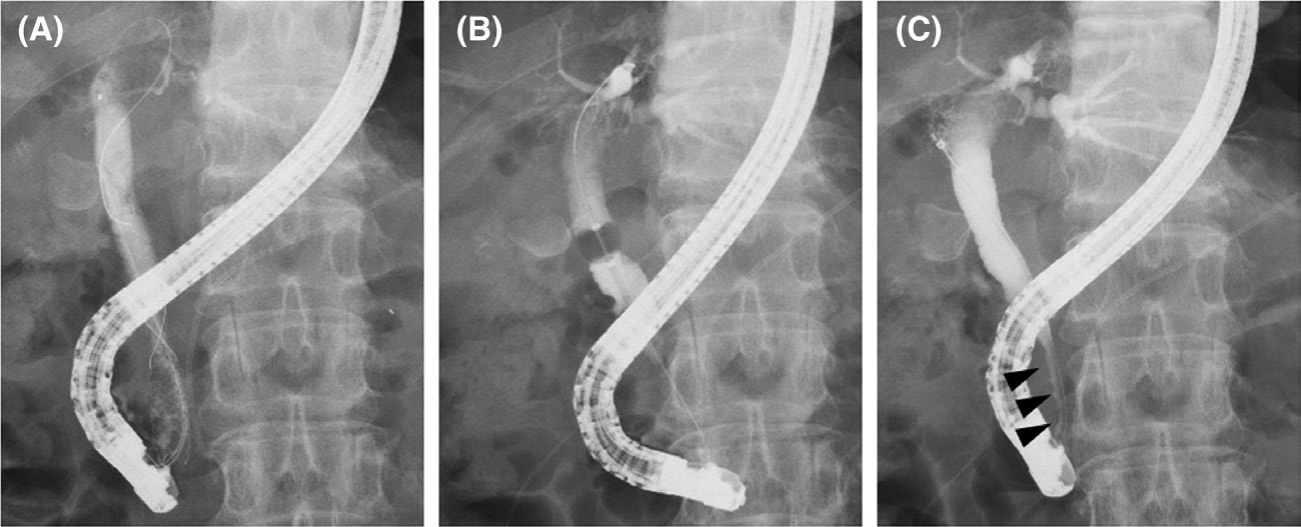
The findings of the second endoscopic cholangiography. A, No stones in the common bile duct (CBD) were observed. B, After the covered self‐expandable metallic stent was removed, the biliary sludge was removed by a balloon catheter. C, It was confirmed that the stricture of distal CBD was improved to some extent (arrowhead)

Endoscopic cholangiography was repeated a week after the first cholangiography. We observed that all the CBD stones detected in the initial cholangiography had disappeared spontaneously (Figure 3A). After the CSEMS removal, the biliary sludge was removed using a balloon catheter (Figure 3B). We confirmed that there were no stones in the CBD, and the stricture of the distal CBD showed slight improvement (Figure 3C). Finally, the pancreatic stent was removed.

Approximately a year after the CBD stones were removed using the CSEMS, no recurrence of the CBD stones was observed on the follow‐up imaging at an outpatient clinic.

## 3 DISCUSSION

There are three reports in the literature about cases of CBD stones with distal CBD strictures that were treated using CSEMS (Table 1). However, two case series did not mention the details of the cases with CBD stones and distal CBD strictures. A case series by Cerefice et al^8^ comprised cases in which the CBD stones were difficult to remove using conventional endoscopic methods. Another case series by García‐Cano et al^10^ comprised older patients who had difficulty in tolerating the endoscopic lithotripsy due to the long duration of the procedure.

**TABLE 1:** Previous reports of CBD stones with distant CBD stricture treated using CSEMS

First author, y	Number of cases with CBD stricture	Reason for CBD stricture	CSEMS	Duration of CSEMS insertion	All stone removal
Cerefice et al, 2011^8^	5	NA	Wallstent or Viabil	NA in patients with distant CBD stricture	
Okabe et al, 2012^9^	1	CP	Wall Flex	2 wk	Achieved by lithotripsy
García‐Cano et al, 2013^10^	11	NA	Wall Flex	90‐389 d	7/11
This case	1	CP	BONASTENT M‐intraductal	7 d	Spontaneously disappeared

Abbreviations: CBD, common bile duct; CP, chronic pancreatitis; CSEMS, covered self‐expandable metallic stent; NA, not available.

A case report by Okabe et al^9^ described a case of CBD stones and distal CBD stricture due to CP. The CBD stones remained in the biliary duct and the upper portion of the CSEMS, and the distal biliary stricture did not improve despite CSEMS placement for 14 days. In contrast, the CBD stones spontaneously disappeared in our case, and the distal biliary stricture also improved slightly after the placement of the CSEMS for only 7 days. This indicates that the new CSEMS BONASTENT M‐intraductal (Standard Sci Tech) can lead to good dilatation. The stent's dumbbell shape was thought to slightly prevent stent migration and transmit a dilation force to the CBD stricture.

The CSEMS should be placed for a longer duration to avoid recurrence of the CBD stones. Although CSEMS dilated the distal CBD stricture, the distal CBD remained thin (Figure 3C). In the previous reports, the duration and effectiveness of the CSEMS placement for CBD strictures secondary to CP were variable. Cahen et al^20^ reported six cases that received CSEMS for CBD stricture with CP. They reported the CSEMS removal time to be 3‐6 months and that 66% of the CBD strictures showed improvement. Lalezari et al^21^ reported a case with CP in which the CBD was dilated by CSEMS. In this report, the CSEMS was placed for 63 days, and the CBD stricture had improved. Haapamäki et al^22^ conducted a randomized controlled study on the effectiveness of multiple plastic stents vs the CSEMS in treating biliary stricture with CP. In this study, CSEMS was removed 6 months after randomization, and the 2‐year stricture‐free success rate was 92% (24/26). Although the targets were not CP patients alone, Park et al^23^ increased the duration of CSEMS placement to ≥120 days for resolution of the CBD stricture.

However, complications such as migration and embedment have been reported when the CSEMS was left in place for a longer duration.^10^, ^20^, ^23^, ^24^, ^25^ A new dumbbell‐shaped CSEMS is thought to be useful in preventing migration. The duodenal tip of the dumbbell shape can prevent the embedment and migration into the biliary tract superiorly, while the hilar tip can prevent duodenal migration. If the new dumbbell‐shaped CSEMS migrates into the biliary tract superiorly, removal of the stent is thought to be easier than removing other types of the CSEMSs, primarily because the dumbbell‐shaped CSEMS becomes narrower by pulling a string that is attached at the duodenal tip (Figure 2). Park et al^23^ described that migration of the CSEMS is a risk factor that prevents the resolution of benign CBD strictures. The dumbbell shape is expected to prevent migration and dilate the CBD stricture effectively. In fact, in this case, the CBD stricture was slightly dilated after CSEMS placement for only 7 days.

In conclusion, the new dumbbell‐shaped CSEMS is efficient in removing the CBD stones with CBD strictures that occur due to CP. The stent might have the potential to prevent positional displacement and contribute to the dilation of the CBD stricture.

## CONFLICT OF INTEREST

None declared.

## AUTHOR CONTRIBUTION

YI, MS, and TT: wrote the paper. RS, NK, HA, YS, HI, YO, JN, MT, MH, TK, RK, and TH: provided clinical advice. HO: reviewed and wrote the paper.

## ETHICAL APPROVAL

Informed consent was obtained from the patient for inclusion in the study. All procedures were approved by the Institutional Review Board of Fukushima Medical University (approved number: 2453).

## Data Availability

The datasets generated and/or analyzed during the current study are available from the corresponding author upon reasonable request.
